# Live-cell super-resolution imaging unconventional dynamics and assemblies of nuclear pore complexes

**DOI:** 10.52601/bpr.2023.230010

**Published:** 2023-08-31

**Authors:** Xianxin Ye, Minzhu Guan, Yaorong Guo, Xiang Liu, Kunhao Wang, Tongsheng Chen, Shiqun Zhao, Liangyi Chen

**Affiliations:** 1 National Biomedical Imaging Center, State Key Laboratory of Membrane Biology, Beijing Key Laboratory of Cardiometabolic Molecular Medicine, Institute of Molecular Medicine, College of Future Technology, Peking University, Beijing 100871, China; 2 PKU-IDG/McGovern Institute for Brain Research, Beijing 100871, China; 3 Key Laboratory of Laser Life Science, Ministry of Education, College of Biophotonics, South China Normal University, Guangzhou 510631, China

**Keywords:** Super-resolution microscopy, Sparse deconvolution, Nuclear pore complexes, Halo-SiR, Dual-color imaging

## Abstract

Super-resolution microscopy has promoted the development of cell biology, but imaging proteins with low copy numbers in cellular structures remains challenging. The limited number of designated proteins within nuclear pore complexes (NPCs) impedes continuous observation in live cells, although they are often used as a standard for evaluating various SR methods. To address this issue, we tagged POM121 with Halo-SiR and imaged it using structured illumination microscopy with sparse deconvolution (Sparse-SIM). Remarkably, POM121-SiR exhibited more than six-fold fluorescence intensity and four-fold enhanced contrast compared to the same protein labeled with tandem-linked mCherry, while showing negligible photo-bleaching during SR imaging for 200 frames. Using this technique, we discovered various types of NPCs, including ring-like and cluster-like structures, and observed dynamic remodeling along with the sequential appearance of different Nup compositions. Overall, Halo-SiR with Sparse-SIM is a potent tool for extended SR imaging of dynamic structures of NPCs in live cells, and it may also help visualize proteins with limited numbers in general.

## INTRODUCTION

Since its introduction in the 1990s, super-resolution (SR) fluorescence microscopy has promoted the development of cell biology by providing more detailed and dynamic views of cellular structures (Lü *et al.* 2021; Zhang *et al.* 2022). However, as all SR methods require collecting more photons to achieve increased resolution (Gu and Ji 2021; Yang *et al.* 2021), the number of fluorophores labeled sets an upper limit for any protein of interest. This limit is correlated with the protein's copy numbers under physiological conditions in live cells. Therefore, cellular structural proteins such as actin and tubulin are relatively easy to image, while functional proteins with limited copies within cellular structures are challenging to visualize. For example, nuclear pore complexes (NPCs) are enormous, eightfold symmetrical proteinaceous assemblies, and generally, the outer diameter of NPCs ranges from approximately 110 to 130 nm, while their height ranges from 50 to 80 nm (Allegretti *et al.* 2020; Bui *et al.* 2013; Eibauer *et al.* 2015). NPCs contain around 30 different proteins, termed nucleoporins (Nups), and are located on the nuclear envelope. They are the main carriers of macromolecular transport between the nucleus and the cytoplasm, and are crucial in regulating various nuclear activities (Ptak *et al.* 2014). NPCs are prone to viral manipulation, and defective NPCs have been associated with human disorders like cancer (Nofrini *et al.* 2016; Rodriguez-Bravo *et al.* 2018). NPCs have been used as molecular rulers in cells to benchmark the resolution of various SR fluorescence imaging techniques (Thevathasan *et al.* 2019), including stimulated emission depletion (STED) (Gottfert *et al.* 2013) and stochastic optical reconstruction microscopy (STORM) (Sellés *et al.* 2017). Although NPCs are composed of more than 550 copies of ~30 different Nups, the number of individual Nups within an NPC is usually between 8 and 32. As resolution often comes at the price of compromised temporal resolution, it is difficult for SR methods to monitor the dynamic changes of NPCs in live cells.

Previously, we developed a computational SR method, a sparse deconvolution algorithm. Combining sparse deconvolution with structured illumination microscopy (Sparse-SIM) achieved millisecond exposures with ~60-nm spatiotemporal resolution and resolved pore structures labeled by Nups-GFP in live cells (Zhao *et al.* 2022). However, the limited brightness and photostability of green fluorescent protein (GFP) prevent routine long-term imaging of NPCs in live cells. Although GFP and its spectrum variants are widely used (Uno *et al.* 2015), their photophysical properties are generally inferior to organic dyes, emitting photons fewer by one or two orders of magnitude (Dempsey *et al.* 2011; Fernandez-Suarez and Ting 2008). To highlight NPCs better, researchers often use tandem-linked GFP/mCherry, which is also at risk of forming non-physiological aggregations (Daigle *et al.* 2001; Rabut *et al.* 2004). Alternatively, hybrid labeling systems, such as SNAPTag and HaloTag, have been developed to take advantage of the convenience of genetic labeling and brighter organic dyes (Bottanelli *et al.* 2016; Hinner and Johnsson 2010). HaloTag is a self-labeling protein derived from the haloalkane dehalogenase and covalently binds to synthetic ligands (Los *et al.* 2008). The HaloTag can be expressed as a fusion protein with a target protein and selectively reacts with synthetic ligands, such as Halo-SiR and Halo-TMR, which are cell-permeable organic fluorophores with favorable photon properties. Therefore, we labeled NPCs with Halo-SiR, which demonstrated enhanced brightness and a more extended period of SR imaging. Combining Halo-SiR labeling with Sparse-SIM revealed different shapes of nuclear pores, their dynamic interactions, and the sequential appearance of different Nups compositions.

## RESULTS

### Halo-SiR outperforms 3xmCherry in brightness and photostability

**Figure 1:**
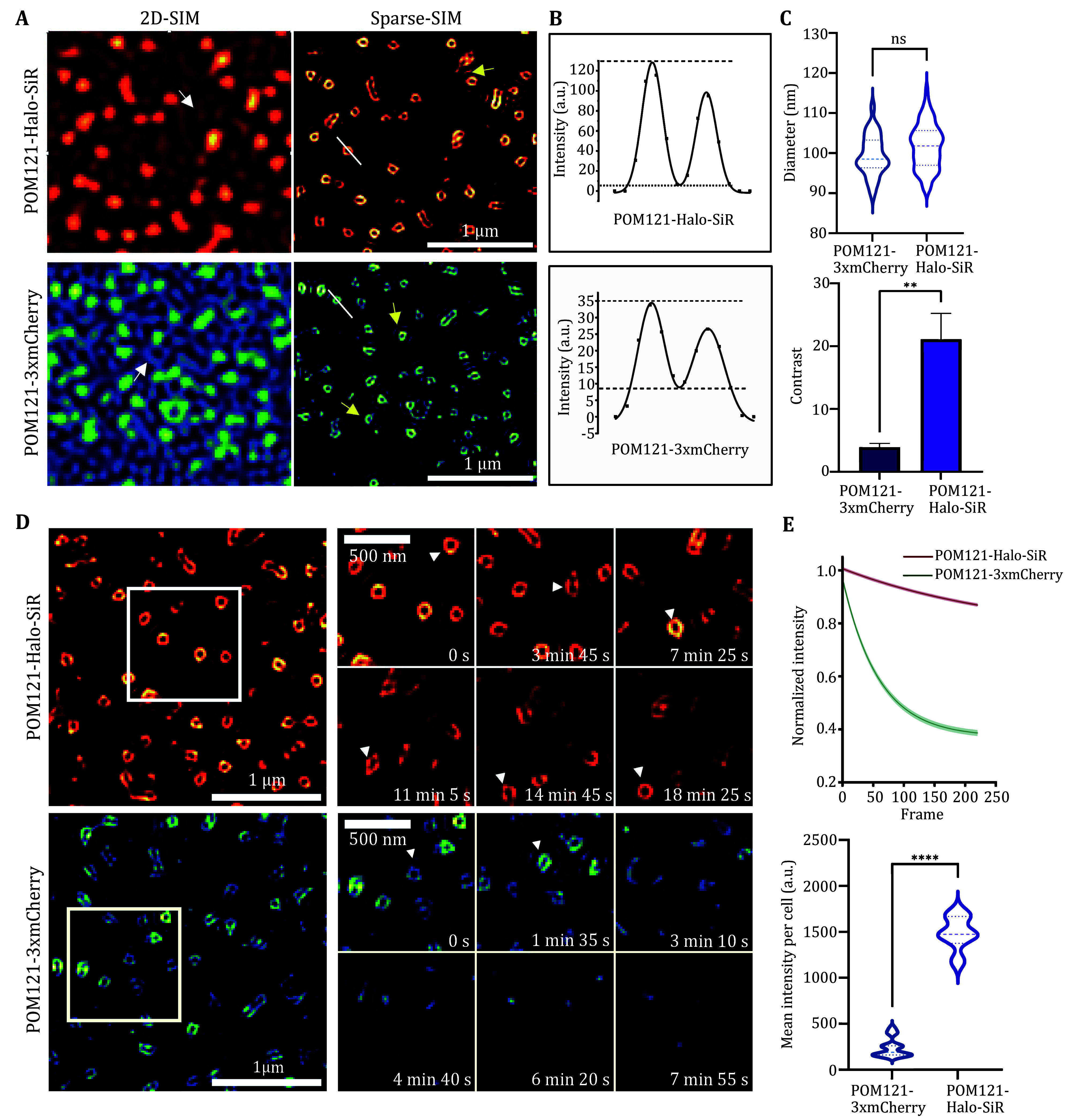


The maximum emission photon flux per second for any live-cell SR imaging systems determines their spatial resolutions. Halo-SiR is conjugated to the near-far-red fluorophore silicon rhodamine (SiR), which exhibits higher quantum yield and photostability than fluorescent proteins. We benchmarked the HaloTag labeling strategy with the 3xmCherry labeling strategy using membrane nucleoporin POM121 as a target, which is present in 8–16 copies in one NPC (Chou *et al.* 2021; Otsuka *et al.* 2023). POM121 fused to either the HaloTag or the 3xmCherry at its C terminus just like before (Dultz and Ellenberg 2010; Dultz *et al.* 2008; Rabut *et al.* 2004). Live MCF7 cells transiently expressed POM121-Halo were incubated with a cell-permeable ligand (Halo-SiR) before imaging. We imaged the two groups under the same conditions in parallel and observed punctuated NPCs under 2D-SIM ( Fig. 1A). A remarkably significant observation is the conspicuous decrease in strip artifacts surrounding the Halo-SiR-labeled nuclear pore complexes, as illustrated in Fig. 1A (indicated by white arrows in the left plane), accompanied by a reduction in deconvolution errors (Culley *et al.* 2018) as depicted in supplementary Fig. S1. After sparse deconvolution to improve the resolution, we obtained ring-like structures of NPCs labeled by either method, similar to those observed under SMLM and STED (Gottfert *et al.* 2013; Uno *et al.* 2014). Furthermore, a notable observation reveals an increased occurrence of ring artifacts surrounding nuclear pore complexes labeled with 3xmCherry in comparison to those labeled with Halo-SiR ( Fig. 1A, indicated by yellow arrows in the right plane). This finding holds substantial importance, indicating a notable improvement in the quality and accuracy of imaging outcomes by using Halo-SiR. The reduction in artifacts and deconvolution errors is indicative of enhanced data fidelity, allowing for clearer visualization and more reliable analysis of the nuclear pore complexes labeled with Halo-SiR. What else, NPCs labeled by Halo-SiR exhibited better contrast compared to those labeled with three tandem mCherry ( Fig. 1A). We plotted the fluorescence intensities of the radial distribution of individual pores before fitting them with Gaussian double peaks, and found that pores of both labels had comparable diameters (~100 nm), but the contrast of Halo-SiR was more than five-fold than that of three tandem mCherry ( Figs. 1B and 1C). Additionally, we conducted time-lapse live-cell SR imaging at intervals of 5 s and found that Halo-SiR enabled extended time-lapse imaging for up to 18 mins, in contrast to the diminished POM121-3xmCherry pores after only ~3 min of recording ( Fig. 1D). Overall, compared to 3xmCherry-labeled NPCs, Halo-SiR-labeled NPCs are brighter and resistant to photo-bleaching and less artifacts under Sparse-SIM, thus leading to more extended observation periods and higher resolution images.

**Figure 1:** Comparison of Halo-SiR and 3xmCherry in contrast and photostability. **A** A representative example of nuclear pores labeled with POM121-Halo or POM121-3xmCherry in a live MCF7 cell. Two-dimensional structured illumination microscopy (2D-SIM) was reconstructed with the Wiener algorithm (left) and Sparse-SIM was reconstructed with the Sparse algorithm (right). **B** Fluorescence intensity profiles of the nuclear pore complex (NPC) labeled by POM121-Halo or POM121-3xmCherry, marked by white lines across the NPC in Panel A. The Gaussian double peaks fitting was performed to calculate the real distance between the peaks, following the protocol outlined in the Methods section. This step was necessary because the diameters of the nuclear pores were comparable to the resolution of Sparse-SIM and the size of the pixel. **C** Violin plot (top) of the diameters of NPCs formed by POM121-3xmCherry ((99 ± 5) nm; *n* = 30 from three cells) and POM121-Halo ((101 ± 5) nm; *n* = 90 from eight cells). The average contrast (peak1 + peak2/2*bottom) of images labeled by 3xmCherry (4 ± 3; *n* = 30 from three cells) and Halo (21 ± 28; *n* = 50 from five cells) were plotted in the bar chart (bottom). **D** Representative montage images labeled by POM121-Halo or POM121-3xmCherry are shown on the top and bottom, respectively. **E**Averaged fluorescence intensities of individual cells are plotted against time (fitting with exponential function, 95% CI, top); violin plot of mean intensity per cell labeled by 3xmCherry (240 ± 102, *n* = 15, bottom) or HaloSiR (1488 ± 185, *n* = 6, bottom). Experiments were repeated independently three times with similar results; *p*-values were calculated using Student’s *t*-test, with * *p* < 0.05, ** *p* < 0.01, **** *p* < 0.0001 indicating statistical significance, and “ns” indicating non-significant results. Error bars represent the standard error of the mean. The original image was captured by HIS-SIM and the imaging interval was 5 s

### NPCs exhibit various morphological dynamics

**Figure 2:**
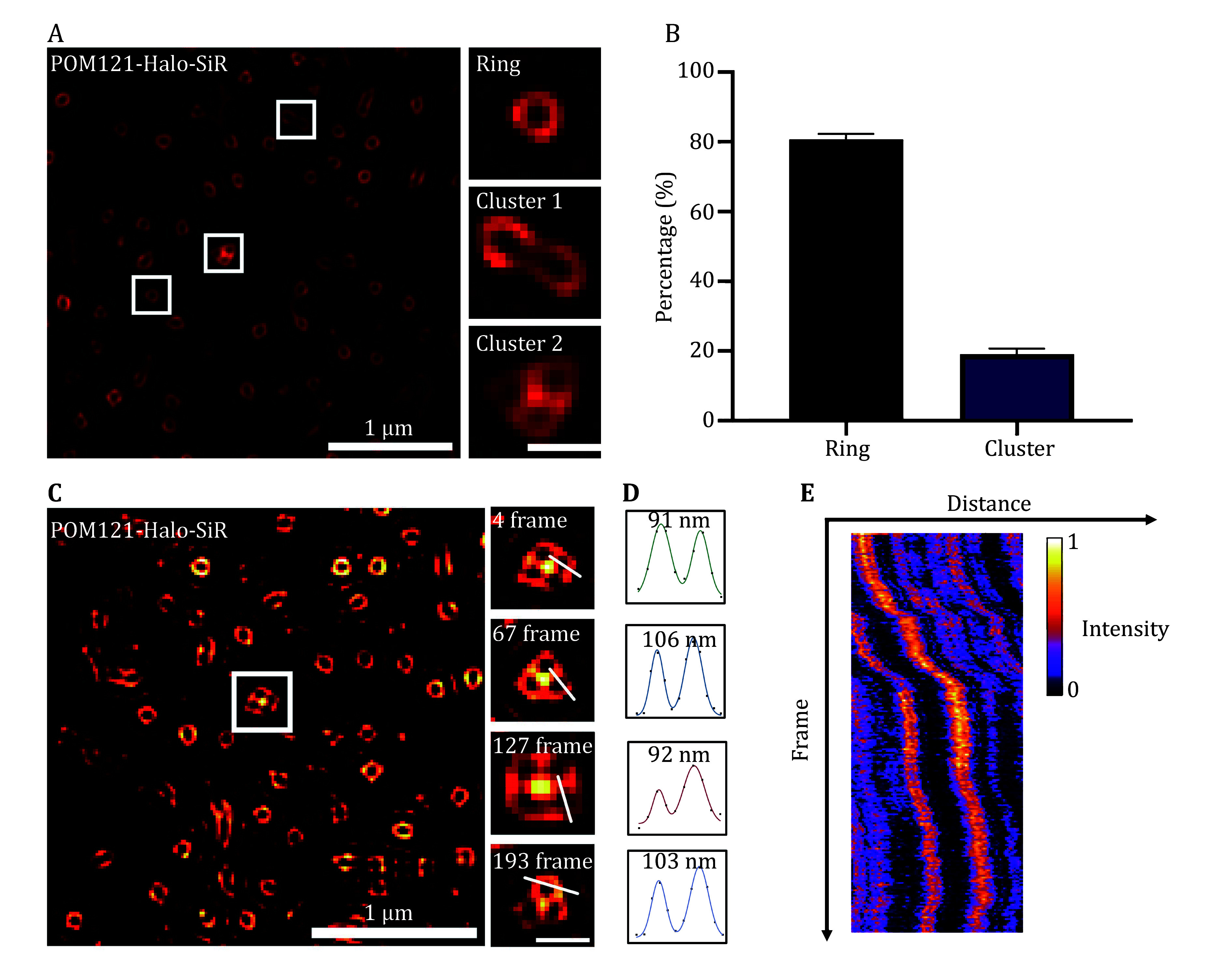
NPCs Morphology and morphological dynamics. **A** A representative example of different nuclear pore structures labeled by POM121-Halo. **B** Percentages of different nuclear pore structures in the images obtained by Sparse-SIM ( *n* = 3 cells). **C** A snapshot of the petaled gathering nuclear pore structure enclosed by a white box (left). The white box on the right is enlarged and shown at four-time points to show the dynamic changes of nuclear pores (right). **D** Intensity profiles and multiple Gaussian fitting of the NPCs are indicated by the straight lines in Panel C (right). Numbers represent the distances between peaks. **E** Kymograph of petaled gathering NPC enclosed by the white box in Panel C (left), showing minimal temporal changes in intensity. Experiments were repeated three times independently with similar results; error bars, SEM. The original image is captured by HIS-SIM, and the imaging frequency is 20 Hz; Scale bars, 200 nm (Panels A and C zoom out)

Next, we utilized the photostable and bright Halo-SiR to investigate the morphological dynamics of NPCs in live cells. Along with the conventional ring-shaped NPCs, we identified irregularly-shaped NPCs, which exhibited linear alignment or petaled gathering ( Fig. 2A，supplementary Videos S1 and S2). Similar behaviors were also observed when NPCs were labeled with fluorescent proteins (supplementary Fig. S2). Although the petaled gathering of multiple nuclear pores has been reported under electron microscopy (Goldberg and Allen 1993; Whytock *et al.* 1990), this minority group of NPCs (20% of the population, Fig. 2B) might have been overlooked in previous SR imaging studies conducted in fixed cells. Interestingly, we observed that the number of individual pores associated with petaled gathering NPCs sometimes changed during long-term imaging ( Fig. 2C), while the total fluorescence intensity of the cluster remained constant ( Figs. 2D and 2E). Notably, we did not observe any temporary separation or insertion of individual pores into the cluster, even with fast temporal sampling (50 ms/frame) (supplementary Video S1). Furthermore, the average diameter of pores was negatively correlated with the number of pores in the cluster ( Figs. 2C and 2D, supplementary Fig. S3). The first class of cluster-like NPCs, which also has been observed under STED and STORM (Gottfert *et al.* 2013; Loschberger *et al.* 2012), represents several NPCs in close proximity below the limiting resolution and exhibits linear alignment due to the membrane topology. These data collectively suggest that NPCs may undergo dynamic remodeling within the nuclear membrane (Sabinina *et al.* 2021).

### Assembly of NPCs from old and newly synthesized nucleoporins

**Figure 3:**
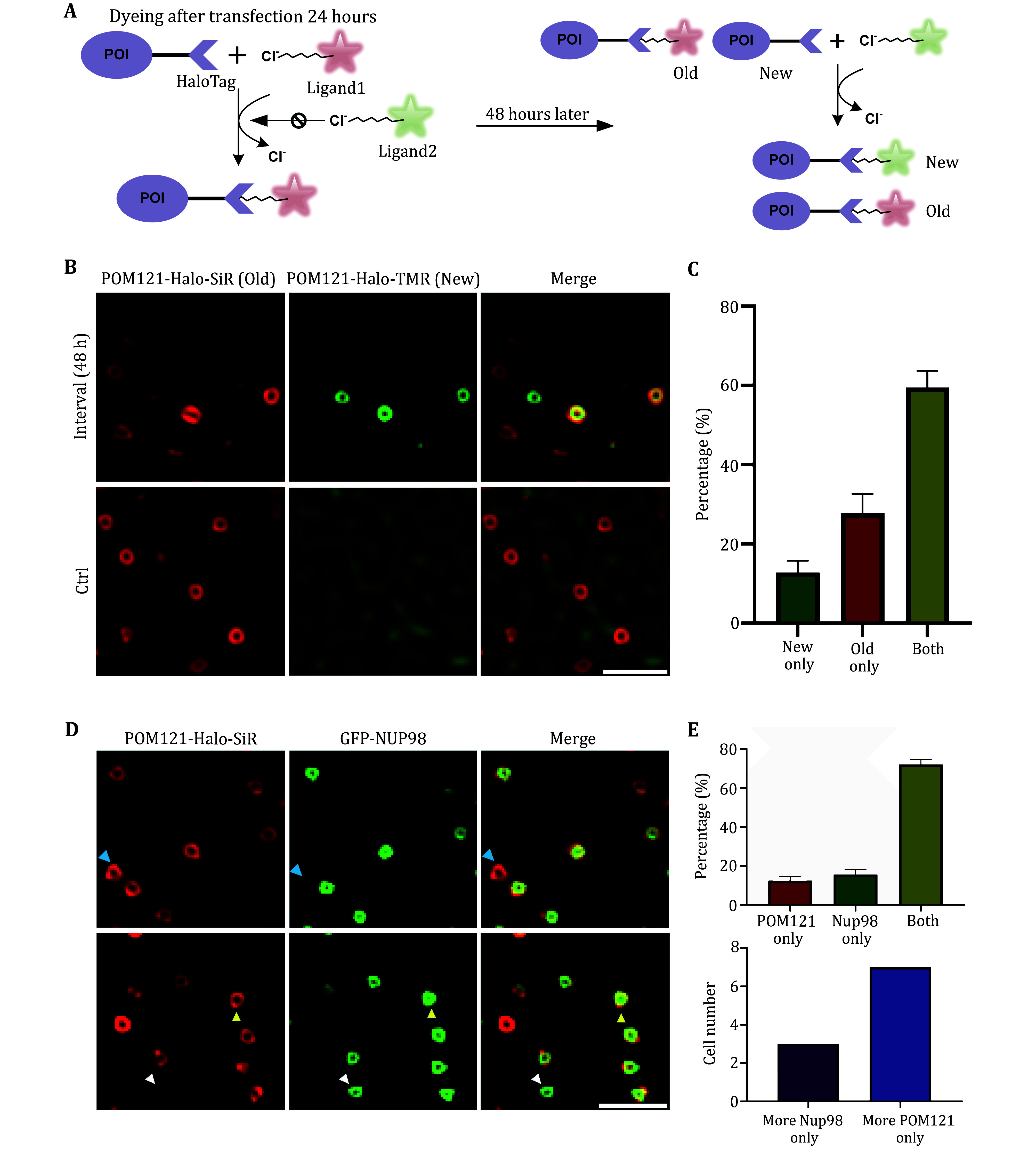
Dual-color imaging reveals different compositions of nuclear pores. **A** Schematic diagram of the experimental pipeline for identifying old and new nuclear pores. **B** A representative example of nuclear pores with different compositions in control and pulse-labeling cells. **C** Statistical results of nuclear pores with different compositions in cells treated ( *n* = 231 from five cells). **D** A dually-labeled nuclear pores by POM121-Halo-SiR and GFP-NUP98. White, cyan, or yellow arrows indicate nuclear pores with GFP-NUP98, POM121-SiR, or both GFP-NUP98 and POM121-SiR, respectively. **E** Percentages of NPCs with different compositions in the images obtained by Sparse-SIM (top, *n* = 1854 from ten cells), and the group of cells with a more selective abundance of POM121 or NUP98 (bottom, *n* = 10 cells). Experiments were repeated three times independently with similar results; Error bars, SEM. Scale bars, 500 nm

The process of depolymerization and reassembly of NPCs in the cell cycle has been studied extensively in recent years (Doucet *et al.* 2010; Dultz and Ellenberg 2010). However, the mechanism by which preexisting and newly synthesized nucleoporins are incorporated into newly assembled NPCs remains unclear. To address this question, we utilized the HaloTag modular protein tagging system, which allows for pulse-labeling of the same fusion protein with different fluorescent dyes. Specifically, we transiently expressed the membrane nucleoporin POM121-Halo and pulse-labeled the cells with Halo-SiR and Halo-TMR to distinguish between old and new POM121 in freshly assembled NPCs. The day after transfection, NPCs are labeled with the Halo-SiR pulse ("Old" POM121, Red). Next, cells were cultured for 48 h and labeled with another round of Halo-TMR pulse ("New" POM121, green) before imaging ( Fig. 3A). Our results indicate that there are three distinct types of nuclear pores in the cell: those with only the TMR signal (representing new pores, 12.7%), those with only the SiR signal (representing old pores, 27.8%), and those with both the SiR and TMR signals (representing mixed pores, 59.4%). These findings suggest that the incorporation of preexisting and newly synthesized nucleoporins into newly assembled NPCs may not be random, and that distinct assembly mechanisms may be at play (Chou *et al.* 2021; Doucet *et al.* 2010; Otsuka and Ellenberg 2018). Further investigations are necessary to determine the functional implications of these distinct nuclear pores and their potential variations in activity.

### Sequential assembly of POM121 and NUP98 in NPCs

The assembly of nuclear pores involves coordinating hundreds of individual proteins, which can be divided into four categories: transmembrane, core scaffold (inner ring and outer ring), linker, and Phe-Gly (FG) (Strambio-De-Castillia *et al.* 2010). The assembly kinetics of different nucleoporins are different, and distinct nucleoporin subcomplexes are sequentially recruited to the nuclear envelope, all contributing to the stereotypic and highly ordered assembly process of NPCs (Doucet *et al.* 2010; Dultz and Ellenberg 2010).

To gain insight into the order in which POM121 and Nup98 are incorporated into forming NPCs, we co-expressed POM121-Halo and Nup98-GFP in MCF7 cells and imaged them. To investigate the sequential assembly of POM121 and NUP98 in nuclear pore complexes, we specifically selected cells that exhibited simultaneous emission of Halo-SiR and GFP signals. This careful selection ensured that the analyzed cell population co-expressed both POM121 and NUP98, enabling us to effectively observe and analyze the assembly process between these two proteins in NPCs. We observed three distinct labeled NPCs: NPCs with labeled POM121 (12%), with labeled NUP98 (16%), and with both labeled POM121 and NUP98 (72%) ( Fig. 3D). As different stages of NPCs assembly contain different nucleoporins (Doucet *et al.* 2010), we hypothesized that the three distinct labeled NPCs represent three NPC intermediates. Notably, of the ten cells we analyzed, seven cells showed a higher proportion of NPCs containing POM121 over that containing Nup98, while three cells had the opposite phenotype ( Fig. 3E). The existence of cells with a higher proportion of Nup98-containing NPCs suggests that Nup98 may be recruited to NPC assembly before POM121 in a small proportion of cells, although most cells are in the interphase stage of the cell population, and interphase NPC assembly events are initiated by the accumulation of POM121, as proposed previously (Doucet *et al.* 2010).

## DISCUSSION

NPCs are crucial for gene regulation (Strambio-De-Castillia *et al.* 2010; Sun *et al.* 2019), and mediate nucleocytoplasmic transport, and morphological alterations in NPCs have been linked to various diseases (Rodriguez-Bravo *et al.* 2018; Sakuma and D'Angelo 2017). However, because each NPC contains a fixed number of specific nucleoporins, live-cell SR imaging of NPCs is severely limited by the photon flux emission rate and fluorophore photostability. To overcome this limitation, researchers have used tandem-tagged versions of GFP/mCherry to label nucleoporins in previous studies (Daigle *et al.* 2001; Rabut *et al.* 2004). In this study, we tagged POM121 with either Halo-SiR or 3xmCherry and used Sparse-SIM to image pore structures of similar sizes. Our results showed that Halo-SiR outperformed 3xmCherry in generating SR images with higher contrast and extended photostability, making it more suitable for imaging other proteins with limited numbers in cells.

The HaloTag is a versatile tool that can be used for pulse-chasing designated biological processes in dual-color or multi-color experiments. In our study, we employed Halo-SiR and Halo-TMR to label live cells and distinguish between new and old Nups and NPCs with different compositions of old and new POM121 (as shown in Fig. 3B). The HaloTag ligands have a remarkable ability to form covalent bonds rapidly, and these bonds are highly specific and essentially irreversible under typical physiological conditions. In our imaging system, the abundance of HaloTag ligands is significantly higher than that of HaloTag proteins. In addition, the most noticeable finding in our experiment is that the control group, which underwent immediate staining with Halo-TMR following Halo-SiR labeling, does not exhibit any NPCs displaying Halo-TMR signals ( Fig. 3B, bottom). The results of another experiment, in which we co-expressed POM121-Halo and POM121-3xmCherry in cells, revealed almost complete co-localization between the two labeling methods (supplementary Fig. S4), indicating that Halo-SiR can naturally bind to Halo-Tag and label nuclear pore complexes efficiently in cells. These results provide robust evidence supporting the hypothesis that nucleoporins exhibiting Halo-TMR signals are newly synthesized. Fast SR imaging at 20 Hz enabled us to observe cluster-like NPCs and atypical morphological changes in NPC clusters (as shown in Fig. 2D). Although the physiological roles of these observations require further study, these results demonstrate the benefits of live-cell SR imaging at extreme spatiotemporal combinations. In the future, we could push the boundaries of multiplexing SR imaging in live cells by replacing GFP with Staygold, a fluorescent protein with exceptional photostability and brightness (Hirano *et al.* 2022).

In summary, our study demonstrates that using the Halo-SiR and Sparse-SIM techniques can reveal intricate protein complex structures at high spatiotemporal resolutions. This finding suggests that these approaches could significantly expand the application of SR imaging methods in live-cell biological studies.

## MATERIALS AND METHODS

### Cell maintenance and preparation

MCF7 cells (ATCC, HTB-22) were cultured in MEM medium (Gibco, 10370021) supplemented with 10% fetal bovine serum (FBS; Gibco) in an incubator at 37 °C with 5% CO _2_. For the 2D-SIM imaging experiments, coverslips (H-LAF 10L glass; reflection index, 1.788; diameter, 26 mm; thickness, 0.15 mm, customized) coated with 10% poly-l-lysine solution (Sigma) for ~24 h before seeding transfected cells.

### Plasmid constructs and synthetic ligands

pPom121-3mCherry (Euroscarf, p30648) was purchase from Euroscarf and pPom121-Halo were created by replacing the three copies of mCherry in pPom121-3mCherry.

### Live-cell imaging

For the 2D-SIM experiments, to label nuclear pores, MCF7 cells were transfected with GFP–Nup98/POM121-Halo. The transfections were executed using Lipofectamine 2000 (ThermoFisher Scientific) according to the manufacturer's instructions. After transfection, cells were plated on precoated coverslips. Cells transiently expressed the POM121-Halo were incubated for 15 min with a cell-permeable ligand (Halo-SiR or Halo-TMR) before imaging. Live cells were imaged in HBSS (Gibco) at 37 °C on the commercialized Hessian-SIM, termed HIS-SIM (High Intelligent and Sensitive Microscope) provided by Guang Zhou Computational Super-resolution Biotech Co., Ltd. Images were acquired using a 100×/1.7 NA oil immersion objective (Olympus). The detailed imaging parameters utilized in this study are described in supplementary Table S1.

### Image analysis

Image processing was performed in ImageJ (National Institutes of Health), and Sparse-SIM uses the sparse deconvolution algorithm following published protocols (Zhao *et al.* 2022).

### Corrections of pore diameters

When the sizes of the system PSF and camera pixels were comparable to the size of the structure being imaged, the fitted diameters of punctated and ring-shaped formations varied from their true values in distinct ways. We corrected these values following the published protocol (Zhao *et al.* 2022)

### Statistical analysis

Statistical analysis was performed using GraphPad Prism 9.0.0. The Student's *t*-test was used to assess differences between the two groups. Statistical significance was defined as * *p* < 0.05, ** *p* < 0.01, **** *p* < 0.0001, and ns, insignificant.

## Conflict of interest

Xianxin Ye, Minzhu Guan, Yaorong Guo, Xiang Liu, Kunhao Wang, Tongsheng Chen, Shiqun Zhao and Liangyi Chen declare that they have no conflict of interest.

## References

[bAllegretti2020] (2020). In-cell architecture of the nuclear pore and snapshots of its turnover. Nature.

[bBottanelli2016] (2016). Two-colour live-cell nanoscale imaging of intracellular targets. Nat Commun.

[bBui2013] (2013). Integrated structural analysis of the human nuclear pore complex scaffold. Cell.

[bChou2021] (2021). Inherited nuclear pore substructures template post-mitotic pore assembly. Dev Cell.

[bCulley2018] (2018). Quantitative mapping and minimization of super-resolution optical imaging artifacts. Nat Methods.

[bDaigle2001] (2001). Nuclear pore complexes form immobile networks and have a very low turnover in live mammalian cells. J Cell Biol.

[bDempsey2011] (2011). Evaluation of fluorophores for optimal performance in localization-based super-resolution imaging. Nat Methods.

[bDoucet2010] (2010). Cell cycle-dependent differences in nuclear pore complex assembly in metazoa. Cell.

[bDultz2010] (2010). Live imaging of single nuclear pores reveals unique assembly kinetics and mechanism in interphase. J Cell Biol.

[bDultz2008] (2008). Systematic kinetic analysis of mitotic dis- and reassembly of the nuclear pore in living cells. J Cell Biol.

[bEibauer2015] (2015). Structure and gating of the nuclear pore complex. Nat Commun.

[bFernandezSuarez2008] (2008). Fluorescent probes for super-resolution imaging in living cells. Nat Rev Mol Cell Biol.

[bGoldberg1993] Goldberg MW, Allen TD (1993) The nuclear pore complex: three-dimensional surface structure revealed by field emission, in-lens scanning electron microscopy, with underlying structure uncovered by proteolysis. J Cell Sci 106 ( Pt 1): 261-274

[bGottfert2013] (2013). Coaligned dual-channel STED nanoscopy and molecular diffusion analysis at 20 nm resolution. Biophys J.

[bGu2021] (2021). Recent progress on single-molecule localization microscopy. Biophysics Reports.

[bHinner2010] (2010). How to obtain labeled proteins and what to do with them. Curr Opin Biotechnol.

[bHirano2022] (2022). A highly photostable and bright green fluorescent protein. Nat Biotechnol.

[bLos2008] (2008). HaloTag: a novel protein labeling technology for cell imaging and protein analysis. ACS Chem Biol.

[bLoschberger2012] Loschberger A, van de Linde S, Dabauvalle MC, Rieger B, Heilemann M, Krohne G, Sauer M (2012) Super-resolution imaging visualizes the eightfold symmetry of gp210 proteins around the nuclear pore complex and resolves the central channel with nanometer resolution. J Cell Sci 125(Pt 3): 570-575

[bL2021] (2021). Live-cell fluorescence imaging of ciliary dynamics. Biophys Rep.

[bNofrini2016] (2016). Nucleoporin genes in human diseases. Eur J Hum Genet.

[bOtsuka2018] (2018). Mechanisms of nuclear pore complex assembly - two different ways of building one molecular machine. FEBS Lett.

[bOtsuka2023] (2023). A quantitative map of nuclear pore assembly reveals two distinct mechanisms. Nature.

[bPtak2014] (2014). The multifunctional nuclear pore complex: a platform for controlling gene expression. Curr Opin Cell Biol.

[bRabut2004] (2004). Mapping the dynamic organization of the nuclear pore complex inside single living cells. Nat Cell Biol.

[bRodriguezBravo2018] (2018). Nuclear Pores Promote Lethal Prostate Cancer by Increasing POM121-Driven E2F1, MYC, and AR Nuclear Import. Cell.

[bSabinina2021] (2021). Three-dimensional superresolution fluorescence microscopy maps the variable molecular architecture of the nuclear pore complex. Mol Biol Cell.

[bSakuma2017] (2017). The roles of the nuclear pore complex in cellular dysfunction, aging and disease. Semin Cell Dev Biol.

[bSells2017] (2017). Nuclear pore complex plasticity during developmental process as revealed by super-resolution microscopy. Sci Rep.

[bStrambioDeCastillia2010] (2010). The nuclear pore complex: bridging nuclear transport and gene regulation. Nat Rev Mol Cell Biol.

[bSun2019] (2019). The Nuclear Pore Complex in Cell Type-Specific Chromatin Structure and Gene Regulation. Trends Genet.

[bThevathasan2019] (2019). Nuclear pores as versatile reference standards for quantitative superresolution microscopy. Nat Methods.

[bUno2014] (2014). A spontaneously blinking fluorophore based on intramolecular spirocyclization for live-cell super-resolution imaging. Nat Chem.

[bUno2015] (2015). A guide to use photocontrollable fluorescent proteins and synthetic smart fluorophores for nanoscopy. Microscopy (Oxf).

[bWhytock1990] Whytock S, Moir RD, Stewart M (1990) Selective digestion of nuclear envelopes from Xenopus oocyte germinal vesicles: possible structural role for the nuclear lamina. J Cell Sci 97 ( Pt 3): 571-580

[bYang2021] (2021). Advancing biological super-resolution microscopy through deep learning: a brief review. Biophys Rep.

[bZhang2022] (2022). Characterization of liquid–liquid phase separation using super-resolution and single-molecule imaging. Biophys Rep.

[bZhao2022] (2022). Sparse deconvolution improves the resolution of live-cell super-resolution fluorescence microscopy. Nat Biotechnol.

